# Neurofilaments as Prognostic Biomarkers in the Assessment of the Risk of Advanced Taxane-Induced Neuropathy in Breast Cancer Patients—A Pilot Study

**DOI:** 10.3390/cancers17060988

**Published:** 2025-03-14

**Authors:** Agata Makówka, Malgorzata Fuksiewicz, Anna Bałata, Anna Borowiec, Katarzyna Pogoda, Zbigniew Nowecki, Agnieszka Jagiello-Gruszfeld, Beata Janas, Beata Kotowicz

**Affiliations:** 1 Cancer Biomarkers and Cytokines Laboratory Unit, Maria Sklodowska-Curie—National Research Institute of Oncology, Roentgena St. 5, 02-781 Warsaw, Poland; 2 Department of Breast Cancer and Reconstructive Surgery, Maria Sklodowska-Curie—National Research Institute of Oncology, Roentgena St. 5, 02-781 Warsaw, Poland; 3 Oncological Clinic I, Maria Sklodowska-Curie—National Research Institute of Oncology, Roentgena St. 5, 02-781 Warsaw, Poland; 4 Maria Sklodowska-Curie—National Research Institute of Oncology, Roentgena St. 5, 02-781 Warsaw, Poland; 5 Occupational Medicine Clinic, Maria Sklodowska-Curie—National Research Institute of Oncology, Roentgena St. 5, 02-781 Warsaw, Poland

**Keywords:** biomarker, NF-L, neuropathy, breast cancer

## Abstract

Objectives: The aim of the present study was to assess the clinical value of measuring the concentration of neurofilament light chains (NF-Ls) in the diagnosis of taxane-induced neuropathy (CIPN) during neoadjuvant chemotherapy (NAC) in breast cancer patients. Methods: This study included a total of 94 patients who qualified for NAC with taxanes. Serum samples were collected before starting NAC, after three and six cycles, and 3–6 months after NAC. The NF-L concentration was determined using the Ella technology. The assessment of CIPN was based on the clinical symptoms included in the EORTC QLQ-CIPN20 scores. Results: The median NF-L concentrations increased during NAT monitoring. After the end of therapy, a significant decrease in NF-L concentrations was observed (*p* = 0.001, R = 0.37). We established a cut-off point of 29.5 pg/mL to distinguish between the control group and patients with early symptoms of neuropathy (CIPN G1) (*p* = 0.001; AUC = 0.982). We showed that NF-L concentrations, regardless of the stage of therapy, increased with the severity of neuropathy symptoms (CIPG1 vs. G2 vs. G3) (*p* = 0.0189, R = 0.33). According to the established cut-off points, serum NF-L concentrations above 196 pg/mL in patients undergoing therapy likely indicate the presence of low-grade neuropathy (*p* = 0.0076), while values above 218 pg/mL may indicate advanced CIPN (*p* = 0.0008). Conclusions: In this study, we demonstrated the usefulness of NF-L levels to confirm neuropathy early in the course of treatment, which is important as the questionnaire-based assessment of neuropathy currently used in practice remains subjective. Ultimately, serum NF-L levels are helpful in determining the severity of NAC-induced neuropathy among breast cancer patients.

## Simple Summary

Data in the literature indicate the significant role of NF-L levels in monitoring polyneuropathy, which may be caused by chemotherapy using taxanes. Previous studies have been conducted in small groups of patients, with the cut-off points and neurofilament measurement schemes used significantly differing. Thus, it is necessary to investigate whether NF-L measurements may become a factor in predicting severe peripheral neuropathy among patients prior to a course of chemotherapy. The determination of NF-Ls during treatment and demonstrating the relationship between NF-L concentrations and the severity of CIPN may serve as a basis for adjusting the dose or changing the drug being administered. Rapid diagnostics with the possibility of assessing one’s predisposition to and the severity of neuropathy would significantly improve both the quality of life and the effects of systemic treatment in patients. Therefore, there is a need to determine an objective and measurable parameter, such as NF-Ls, to define neuropathy.

## 1. Introduction

Taxanes (docetaxel, paclitaxel, and nab-paclitaxel) are cytostatics of natural origin that are commonly used in clinical oncology. For patients suffering from malignant tumors with various locations of the primary lesion, paclitaxel is used in both neoadjuvant and metastatic settings and belongs to a group of drugs with antagonistic effects against microtubules, causing inhibition of the mitosis process in cells [1]. A common side effect of paclitaxel is neurotoxicity due to chemotherapy-induced peripheral neuropathy (CIPN), which is a significant clinical problem [2,3]. CIPN is a side effect of oncological treatment, significantly worsening the quality of life among patients. This condition is often characterized by numbness, tingling, and pain in the limbs. Symptoms may include sensitivity to cold or heat, autonomic and motor dysfunctions, and neuropathic pain that significantly reduces quality of life [4]. Some authors suggest that the increased neuroinflammation associated with chemotherapy in breast cancer is associated with cognitive impairment. This side effect impacts as many as 70% of patients, deteriorating their memory and attention [5]. The overall incidence of neuropathy in patients receiving chemotherapy has been estimated to be 48%, and the risk of CIPN increases to 87% when the patient is administered taxanes [6]. Consequently, patients often need to reduce the dose or even change their therapy, which is associated with a risk of decreased anticancer effectiveness. Biochemical and cellular changes caused by chemotherapy may also be chronic. In approximately 30% of patients, symptoms of neuropathy persist 6 months after the end of systemic treatment (especially after treatment with taxanes and platinum derivatives), and the reversibility of neurotoxicity remains controversial.

It is suggested that the symptoms of neuropathy are a result of the interaction of paclitaxel with TLR-4 (toll-like receptor 4) on macrophages and the activation of a cascade of pro-inflammatory factors. Stimulated immune cells (T lymphocytes, monocytes, neutrophils, and macrophages) move towards peripheral nerves and dorsal root ganglia (DRG), increasing inflammation. In later stages, oxidative stress and the formation of reactive oxygen species lead to intracellular damage. Changes in the excitability threshold of peripheral neurons can also be observed, with the degree of damage to the nervous system being related to the size and time of drug infusion [7,8]. In clinical practice, a combination therapy of paclitaxel and carboplatin is often used, which may further increase the proportion of patients with severe peripheral neuropathy. Symptoms of neuropathy may appear after the first treatment period and worsen after subsequent cycles.

For the purposes of clinical practice, the symptoms of neuropathy have recently been standardized using a symptom severity assessment scale. The National Cancer Institute Common Terminology Criteria for Adverse Events developed a five-level scale, from G1 to G5, with divisions depending on the intensity of discomfort felt by patients [9]. Grade G1 involves paresthesia and tingling without impairment of functioning; G2 entails paresthesia and sensory disturbances impairing functioning, without affecting the patient’s activity; G3 involves sensory disturbances or paresthesia changing the patient’s activity; G4 involves permanent impairment of functioning; and G5 means death. Moderate neuropathy (G1 to G2) is associated with a possible reduction in the dose of the drug. Conversely, G3-G4, a clinically relevant group, suggests the need to change the dose or terminate therapy, while grade G5 concerns death related to the toxic effects of the treatment. Due to an increase in the incidence of malignant tumors, the frequent diagnosis of breast cancer at a young age, and a significant extension of survival time, CIPN is increasingly observed among patients who have to struggle with pain and bothersome ailments [10].

At present, there are no neuroprotective agents to prevent the development of CIPN and no effective treatment options [11]. The known lack of useful methods for treating neuropathy and the condition’s impact on the effects of anticancer therapy have inspired researchers to explore diagnostic methods which can indicate drug neurotoxicity in patients, even before the appearance of clinical symptoms [12,13].

The introduction of new, ultrasensitive research methods in recent years has significantly expanded diagnostic prospects in clinical oncology through the possibility of assessing a wide panel of specific biomarkers in both tissue and serum. Promising biomarkers for neurotoxicity testing include neurofilament light chains (NF-Ls), the high prognostic and diagnostic accuracy of which has been previously determined for some neurodegenerative diseases. Significantly higher concentrations of NF-Ls have been observed in the sera of patients with CIPN [14,15]. Moreover, NF-L concentrations were observed to increase depending on the dose of chemotherapy in breast cancer patients, suggesting their potential as a marker of neuronal damage after chemotherapy [16].

Neurofilaments are components of the cytoskeletons of peripheral neurons and the central nervous system. Taking into account their molecular weight, we can distinguish three subunits: NF-Ls (light chains), NF-Ms (medium chains), and NF-Hs (heavy chains). Neurofilaments are involved in the growth of axons, maintaining their stability, as well as that of mitochondria [17]. Gradual damage to neuronal cells caused by injury to the peripheral nervous system in chemotherapy-induced peripheral neuropathy leads to the release of neurofilaments, primarily NF-Ls. This process leads to an increase in their concentrations in the cerebrospinal fluid. Then, these neurofilaments are removed into the bloodstream via drainage along the basement membranes of the arteries through the cervical/lumbar lymph nodes [18].

The first studies on the determination of NF-Ls in patients focused on those diagnosed with a neurodegenerative disease. For example, the usefulness of NF-L tests in monitoring the course of Alzheimer’s disease was previously demonstrated. An increase in the concentration of NF light chains signaled the progression of the disease, even before the onset of clinical symptoms [19]. The usefulness of NF-Ls in monitoring multiple sclerosis and amyotrophic lateral sclerosis patients has also been confirmed [20,21]. Notably, NF-L levels may vary according to physiological and pathological factors. Higher levels of NF-Ls were previously observed in older individuals and those with renal failure, regardless of age and other factors [22].

In this study, we sought to determine the clinical value of measuring the concentration of neurofilament light chains (NF-Ls) in the diagnosis of taxane-induced neuropathy in the treatment of breast cancer patients.

## 2. Materials and Methods

This study included a total of 94 patients with early breast cancer who qualified for paclitaxel therapy, including 58% with luminal (including 29% luminal A and 71% luminal B) and 41% with triple-negative type cancer (TNBC). The examined patients were between 27 and 77 years old (median 50 years). The control group consisted of 42 healthy women who were employees of the Maria Skłodowska-Curie National Research Institute of Oncology, aged 22 to 69 years (median age 43 years), with no history of neuropathy, depression, or addiction to psychoactive substances. Serum samples were collected (1) before starting neoadjuvant chemotherapy based on taxanes, (2) after three and six treatment cycles, and (3) 3–6 months after completing NAC. The clinicopathological characteristics of the patients included in this study are presented in Table 1.

Consent was obtained from the Bioethics Committee at the Maria Skłodowska-Curie National Research Institute of Oncology No. 3/2023.

Blood samples were centrifuged, and the obtained sera were stored in a low-temperature freezer until testing. The NF-L concentrations in all serum samples were determined with BioTechne kits (Minneapolis, MN, USA) using Ella technology. The Ella analyzer performs immunoenzymatic reactions in a microfluidic cartridge that contains all the necessary reagents for the detection of NF-Ls in serum samples. The sensitivity of the Ella NF-L detection assay was 2.7 pg/mL.

The assessment of CIPN was based on clinical symptoms included in EORTC QLQ-CIPN20 scores (EORTC Quality of Life Questionnaire (CIPN 20)), consisting of 19 questions regarding characteristic symptoms of chemotherapy-induced neuropathy depending on their severity. The CIPN assessment consisted of two components—the patient’s assessment of symptoms (EORTC QLQ-CIPN20 score) and the physician’s assessment according to the National Cancer Institute Common Terminology Criteria. This dual method of assessing the severity of CIPN was chosen to enable both the patient’s and the healthcare professional’s assessment of CIPN for accurate analysis in further publications. Few authors have undertaken similar solutions [23].

The presence of neuropathy was assessed and analyzed by the physician based on a questionnaire completed before starting NAC with paclitaxel, after cycles three and six, and after the end of treatment. The patients did not implement any strategies for neuropathy prevention during chemotherapy. The NCI-CTCAE (National Cancer Institute—Common Terminology Criteria for Adverse Events) scale version 5.0 was used for this assessment, with symptoms ranging from G1 to G4. At the time of enrollment in this study, the presence of neuropathy unrelated to paclitaxel chemotherapy was also assessed in each patient based on possible baseline symptoms. The STATISTICA 9.0 program (StatSoft) was used for statistical calculations. Nonparametric tests such as the Mann–Whitney U-test, Kruskal–Wallis test, and Spearman rank correlation coefficient were used. The diagnostic power of the determined parameters was analyzed using the MedCalc v8.1 for Windows software. Receiver operating characteristic (ROC) analysis was used to determine cut-off points for the tested parameters depending on the severity of neuropathy. The prognostic value was performed using the Kaplan–Meier method, applying one-way analysis with a log-rank test to compare survival curves.

## 3. Results

### 3.1. Clinical Symptoms of Neuropathy During NAC Treatment

According to the data from the CIPN20 questionnaire, 34 (36%) patients were diagnosed with CIPN during treatment with taxanes (NAC). Based on the most frequently reported symptoms of neuropathy (i.e., tingling in the hands or feet or burning in the hands and feet), we observed that the first symptoms appeared in patients after the third weekly cycle of therapy with paclitaxel. The most frequently reported feeling was tingling in the feet. This symptom occurred in half of the respondents. Additionally, 25% of patients reported mild, burning pain in their hands and feet. For most patients, the intensity of symptoms was constant. The symptoms of 13 patients whose polyneuropathy developed to a significant degree (according to the NCI-CTCAE G2 and G3 scale) persisted long after the end of chemotherapy, indicating a chronic condition.

### 3.2. Dependence of Neurotoxicity and NF-L Concentrations on NAC Treatment Regimen: PCL vs. PCL Plus CARBO

According to the assumptions of this study, all patients received potentially neurotoxic chemotherapy with various regimens and sequences, including 61 patients who received paclitaxel (PCL) alone, i.e., one drug with neurotoxic effects. In total, 33 patients received NAC in the PCL plus carbo regimen (carboplatin with a possible neurotoxic effect). The use of another NAC regimen (i.e., AC-doxorubicin and pemrolizumab) presented no clear association with neurotoxicity, nor did the use of dose-dense administration every 2 or 3 weeks. Moreover, the administration of trastuzumab alone, in principle, should not affect CIPN. The Mann–Whitney analysis did not show significant differences in NF-L concentrations between the group of patients treated with the NAC sequence containing neurotoxic PCL and the group treated with PCL plus carbo (*p* > 0.05). Only a slight increase in the percentage of CIPN was observed after the sixth cycle of NAC in patients treated with PCL plus carboplatin. Table 2 shows the percentage of patients with CIPN symptoms and median NF-L concentrations at subsequent stages of NAC, depending on the NAC regimen based on taxanes (PCL vs. PCL plus carboplatin; Table 2).

### 3.3. Analysis of NF-L Concentrations During NAC, Regardless of CIPN Grade

**Figure 1:**
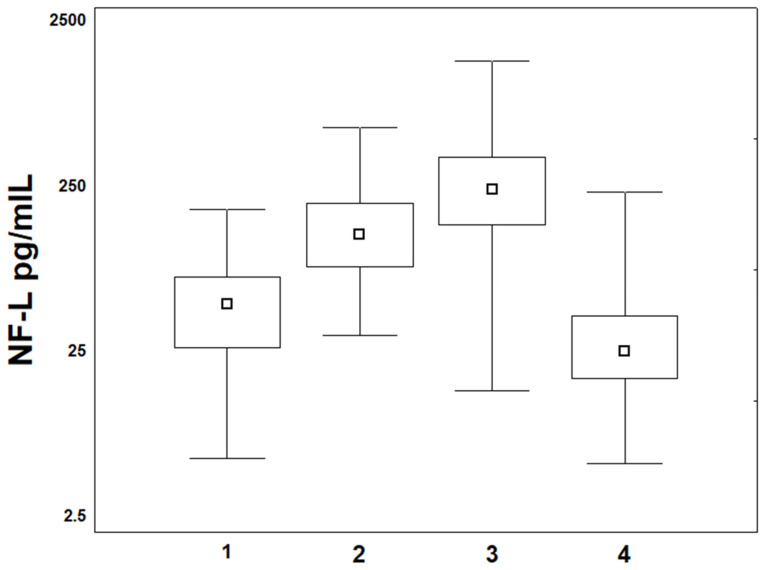
Ranges and median NF-L concentrations before treatment (1), after 3 weekly cycles (2), after 6 cycles (3), and 3–6 months after completion of NAC (4) in breast cancer patients.

The median NF-L concentrations in the control group were significantly lower than those in the sera of patients before the start of paclitaxel therapy and amounted to 6.75 pg/mL, while the median in patients was 47.6 pg/mL. The Mann–Whitney test showed significantly higher NF-L concentrations in the patient group than in the control group before treatment (*p* = 0.001). However, no correlation was found between NF-L concentrations and clinicopathological features, namely, tumor size (T), lymph node status (N), tumor grade (G), biological subtype of breast cancer, and Ki67. The median NF-L concentrations increased during NAC monitoring. After three weekly paclitaxel+/− carboplatin cycles, the median was 127 pg/mL. After six treatment cycles, the median was 236 pg/mL. However, after the end of therapy, a significant decrease in NF-L concentrations was observed (median 24.8 pg/mL). The Kruskal–Wallis test showed no statistical differences between the results of NF-L concentrations in the patient group at our specific NAC times (see the “Materials and Methods” Section) (*p* = 0.001, R = 0.37) (Figure 1). Before treatment, the univariate log-rank analysis did not reveal any prognostic value for NF-L concentrations in determining the occurrence of CIPN during NAC (*p* > 0.05).

### 3.4. Analysis of the Relationship Between NF-L Concentrations and the Stage of CIPN Advancement According to the NCI-CTCAE Scale in Patients Undergoing NAC

The grade of CIPN (G0-G3) was determined in patients during treatment and 3–6 months after the completion of therapy. It was not possible to collect blood from all patients for testing during follow-up. In seven patients, the occurrence of neuropathy unrelated to treatment was observed before the start of therapy with taxanes. The median NF-L concentration was slightly higher (63.1 pg/mL) in this group than among the remaining patients (43.9 pg/mL).

We observed that, during NAC using taxanes, the number of patients with higher-grade neuropathy (CIPN G2 and CIPN G3) increased. After the third paclitaxel cycle, neuropathy was observed in 23 patients (36%), while after six cycles, neuropathy was found in 32 patients, i.e., 52%. CIPN G1 neuropathy predominated after both the third and sixth cycles of treatment. During treatment, the symptoms of neurotoxicity intensified, and the number of patients with advanced CIPN G2 and CIPN G3 increased. Three to six months after completing treatment, G1, G2, and G3 neuropathies were observed in 24 patients (42%). As before, we confirmed that the median NF-L concentrations increased during NAC and significantly decreased at 3–6 months after the end of treatment. This result, however, did not correspond to the grades of CIPN according to the NCI-CTCAE scale (higher medians with lower CIPN grades) (Table 3).

### 3.5. Assessment of the Diagnostic Value of NF-L Levels in Differentiating the Stage of Taxane-Induced CIPN

**Figure 2:**
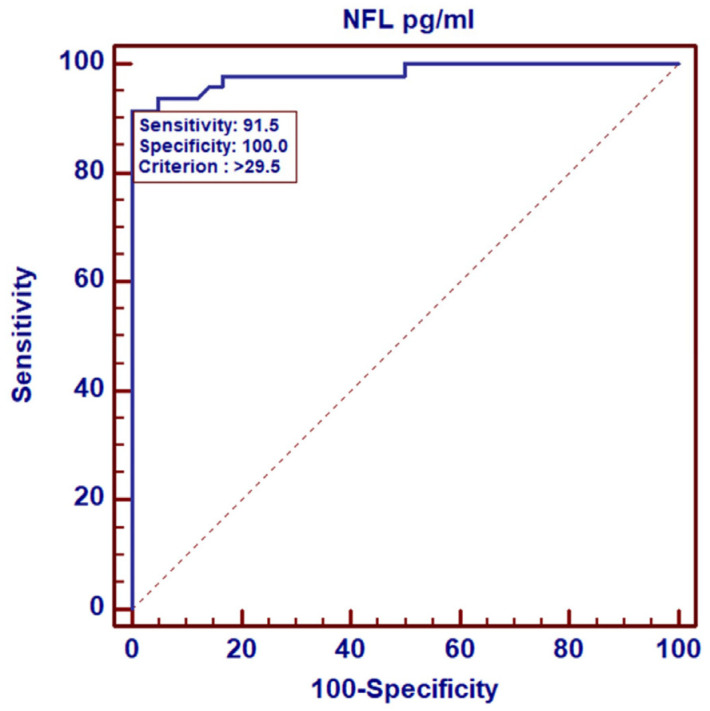
ROC curve for NF-L concentrations in the control group compared to concentrations in breast cancer patients with CIPN G1.

**Figure 3:**
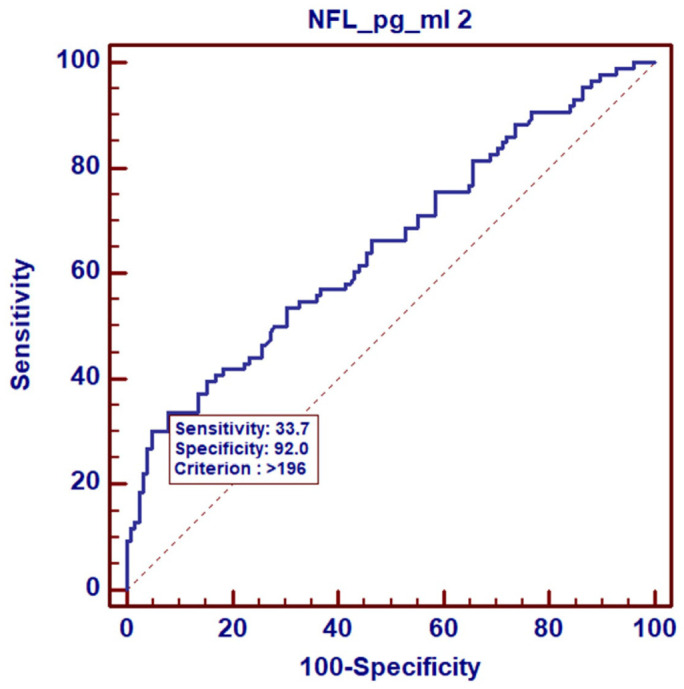
ROC curve for NF-L levels in breast cancer patients without neuropathy, CIPN G0 vs. patients with CIPN G1.

**Figure 4:**
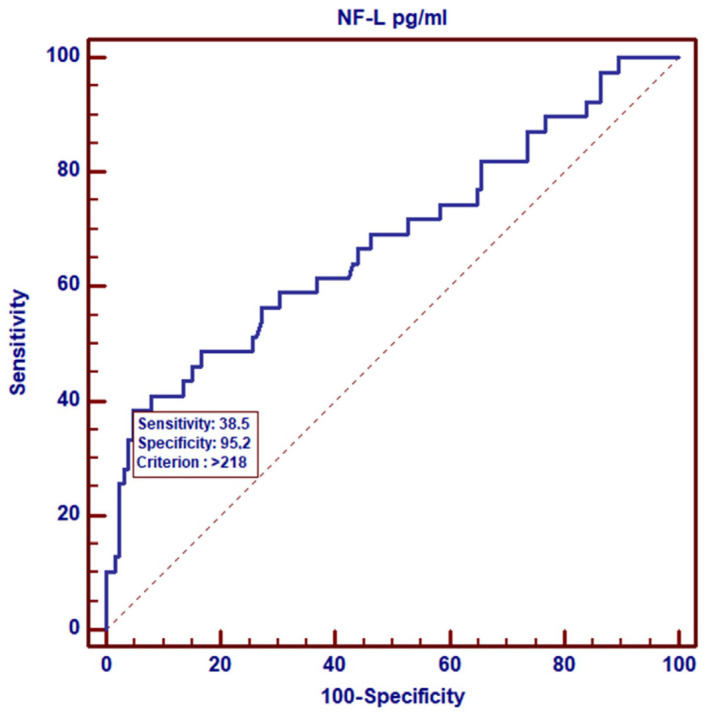
ROC curve for NF-L levels in patients with CIPN G0 vs. patients with CIPN G2 and CIPN G3.

Based on the ROC curves, we analyzed the NF-L levels in the control group of healthy women vs. breast cancer patients with early symptoms of neuropathy (CIPN G1). Regardless of the treatment point, the results indicate that the diagnostic sensitivity for NF-Ls was 100% with a specificity of 91.5% at the cut-off point of 29.5 pg/mL, *p* = 0.0001. The area under the ROC curve (AUC) was 0.982 (95% CI-0.928–0.999) (Figure 2). Analyzing NF-L levels only in the group of patients, i.e., in patients without symptoms of neuropathy (G0) vs. patients with low-grade neuropathy (G1), we observed a smaller area under the curve, AUC = 0.630 (95% CI-0.553–0.702), with a cut-off point of 196 pg/mL and a significance level of *p* = 0.0076 (Figure 3). However, the analysis of NF-L concentrations in CIPN G0 patients vs. patients with higher-grade neuropathies CIPN G2 and CIPN G3 showed that the area under the curve (AUC) was similar at 0.681 (95% CI-0.603–0.751), with a slightly higher cut-off point of 218 pg/mL and a significance level of *p* = 0.0008 (Figure 4).

**Figure 5:**
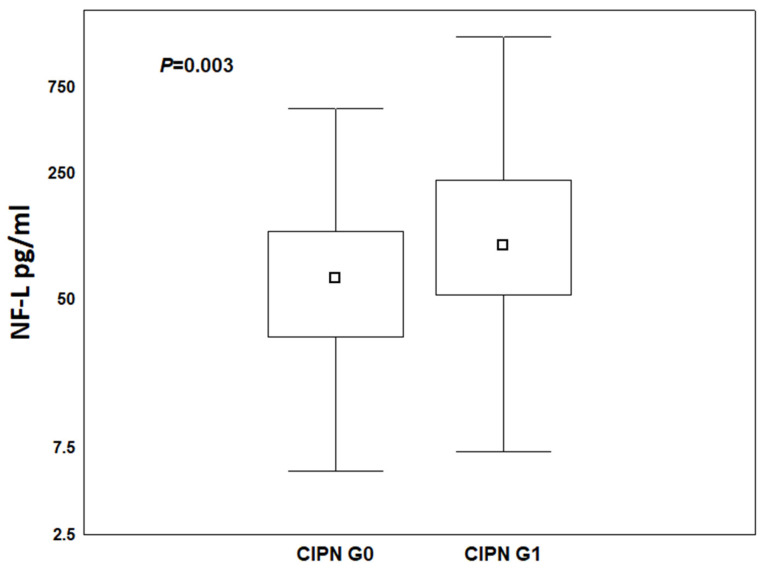
NF-L concentration ranges and medians in patients treated with NAC with paclitaxel without symptoms of neuropathy, CIPN G0 vs. CIPN G1.

Moreover, comparing the median NF-L concentrations regardless of the treatment period, the Mann–Whitney test confirmed significantly higher NF-L concentrations in patients with CIPN G1 vs. those with CIPN G0 (*p* = 0.003) (Figure 5).

**Figure 6:**
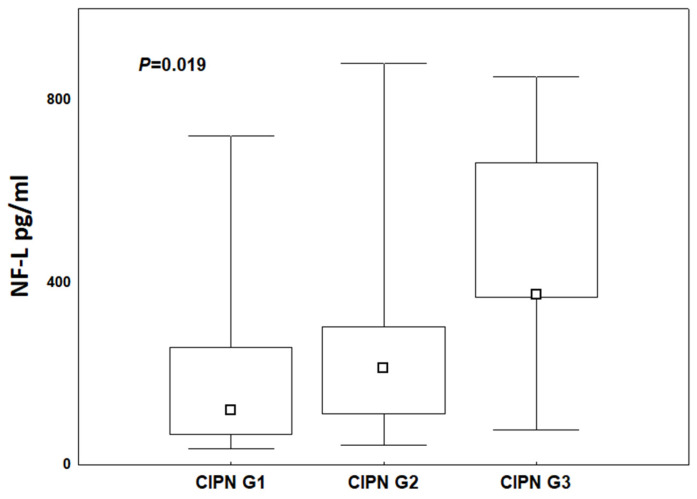
NF-L concentration ranges and medians in patients treated with NAC with taxanes in CIPN grades: CIPN G1 vs. CIPN G2 vs. CIPN G3.

Using the Kruskal–Wallis test, we showed that NF-L concentrations, regardless of the stage of therapy, increased with the severity of neuropathy symptoms (CIPN G1 vs. CIPN G2 vs. CIPN G3) (Figure 6). The differences in concentrations were statistically confirmed (*p* = 0.0189, R = 0.33).

## 4. Discussion

Most of the recent literature has focused on NF-Ls in the context of neurodegenerative diseases. However, there are relatively few studies in the field of oncology and neurotoxicity, and the available results are ambiguous. The authors of these studies indicated the need for validation and verification of their results. Our study is unique as it includes a homogeneous group of patients with breast cancer treated perioperatively, according to the standards of care in a single oncology center. Moreover, there is no objective parameter for assessing chemotherapy-induced polyneuropathy, and the problem of neurotoxicity under systemic treatment in oncology remains topical and important.

Taxane-induced peripheral neuropathy is a barrier to effective cancer treatment and affects the quality of life of patients. Our results confirm previous reports that patients with acquired peripheral neuropathies show elevated serum NF-L levels compared to the control group without neurological diseases. Determination of NF-L levels during treatment and demonstrating the relationship between NF-L levels and the severity of CIPN may enable dose adjustment or drug changes. Elevated levels of NF-Ls should prompt neurological examination. Serial measurements with decreasing or increasing titers may be useful for predicting recovery or deterioration of neurological conditions. Rapid diagnostics with the ability to estimate the severity of neuropathy would certainly improve both the quality of life of patients and the effects of systemic treatment. Therefore, early detection of the first symptoms and appropriate responses are extremely important measures to protect patients from permanent deterioration of their quality of life.

To date, few studies have measured NF-L levels in breast cancer patients with neuropathies resulting from the use of taxanes during NAC therapy. Our study included breast cancer patients treated with paclitaxel. The neuropathy symptoms of these patients were assessed according to NCI-CTCAE grades using the EORTC QLQ-CIPN20 questionnaire during NAC therapy monitoring [24]. The severity of neurotoxicity symptoms after taxane administration during therapy was also assessed. The occurrence of neuropathy after the third and sixth treatment cycles was observed in 36% and 52% of patients, respectively. Following the sixth three-week treatment cycle, CIPN 1 predominated. However, neurotoxicity symptoms worsened during treatment, and the number of patients with significantly advanced CIPN G2 and CIPN G3 increased. We estimated that CIPN affects up to 70% of patients treated with paclitaxel, about 30% of whom experience severe symptoms [25]. CIPN was observed by Brady in 42.7% of cases in a study on patients treated with intravenous paclitaxel and nab-paclitaxel [26].

Our observations showed that after the sixth cycle of treatment, a higher percentage of observed neuropathy cases (58%) occurred in patients treated with paclitaxel and carboplatin compared to those treated with paclitaxel alone (49%). This result suggests greater neurotoxicity when paclitaxel is used in combination with carboplatin. On the other hand, data from the literature indicate a small difference in the toxicity of paclitaxel and cisplatin/carboplatin in combination with paclitaxel (CIPN in 70.8% and 73.0% of patients, respectively) [27]. In our study, NF-L levels were measured in serum samples collected from patients during treatment at four time points: before starting therapy, after three or six cycles of taxane administration, and three to six months after the end of treatment. Before starting therapy, we observed higher NF-L sera levels among patients compared to those among healthy individuals. However, in the study group, we did not observe statistical correlations between NF-L levels and the selected clinicopathological features of breast cancer (tumor stage, presence of lymph node metastases, biological type, and Ki67), which suggests the existence of additional causes of damage to the nervous system associated with the presence of the tumor. Some imaging studies in the literature showed that elevated NF-L levels can be detected before brain metastases [28,29]. Neurological disorders in cancer patients may also be due to inflammation caused by the invasion of tumor cells into the meninges or autoimmune reactions [30]. 

In our observations, the median serum NF-L levels in patients increased during NAC monitoring and decreased after its completion, similar to the observations of other investigators. Kim et al. showed a mild increase in NF-L levels between baseline and 3 months of treatment in a cohort of 34 cancer patients treated with a paclitaxel-based regimen (median = 22.3 pg/mL); however, more pronounced changes were observed between 3 and 6 months of chemotherapy (median = 115.0 pg/mL) [31]. Significant decreases in serum NF-L levels were observed after the end of therapy.

Similar studies by Karteri et al. also demonstrated the usefulness of NF-L levels in monitoring peripheral neuropathy in patients with breast cancer [32]. The results were obtained from NF-L measurements of 59 patients undergoing 12 weeks of paclitaxel chemotherapy. Serum NF-L levels were measured before treatment and after 2, 3, and 12 weeks of treatment. Based on the obtained results, NF-L levels at week 3 of treatment were clinically useful, and levels above 85 pg/mL had a prognostic value for CIPN G2 and CIPN G3 at week 12 of chemotherapy. Our analysis did not show the prognostic value of pre-treatment NF-L levels for determining the occurrence of CIPN during NAC therapy. For this reason, our study required us to expand the number of patients observed in each treatment period.

The median NF-L levels in our control group were significantly lower than those in the sera of patients before the start of therapy, which emphasizes the potential role of NF-Ls as an early biomarker for subclinical CIPN. We showed the usefulness of NF-L measurements as a confirmatory test for the presence of early-stage neuropathy, which is extremely important because symptoms assessed only via a questionnaire are subjective. However, the significant increase in NF-L levels among patients with CIPN G1 compared to the levels among healthy individuals clearly indicates the toxicity of drugs administered in the early phases of taxane treatment.

NF-Ls seem to be a promising marker for monitoring neuropathy, providing useful information on the severity of neuropathy. We showed that NF-L levels increased with the severity of neuropathic symptoms. We observed significant differences in NF-L levels in the group of patients with CIPN neuropathies of various severity (G1, G2, and G3 and patients without neuropathy), regardless of the observation period. However, in contrast to the work of other researchers, we did not observe a strict correlation between NF-L levels and the severity of CIPN in the individual phases of chemotherapy [33,34]. In addition, we found that NF-L levels were significantly higher among patients with significant neuropathy (i.e., CIPN G2 and CIPN G3) compared to those in patients with CIPN G1. This result clearly highlights the potential role of serum NF-L measurements in patients as a biomarker for CIPN during treatment. In other studies, increases in NF-L levels were shown to correlate with the clinical severity of CIPN during paclitaxel-based chemotherapy [34]. A median NF-L concentration of 509 pg/mL was observed among gynecological patients treated with paclitaxel [35]. Similarly, in our study, the median NF-L levels were the highest after cycle six, with 662 pg/mL for CIPN G3. We observed a significant decrease in NF-L levels 3–6 months after the end of NAC therapy. In our observations, however, this decrease in NF-L levels did not correspond to the CIPN grades assessed via the NCI-CTCAE scale; after the end of therapy, patients showed significant G2 and G3 neuropathy. Similar results were obtained in other experiments that treated patients with, among other drugs, paclitaxel. In these studies, persistent neuropathies did not reflect the measured levels of NF-Ls [34]. According to data in the literature, the severity of CIPN usually decreases after the end of chemotherapy, but it can also be chronic in patients with other severe diseases [36]. This was observed in 58–64% of breast cancer patients who completed chemotherapy within 5 years and were treated with taxanes [37].

The next objective of our study was to assess the diagnostic value of NF-L concentration measurements in breast cancer patients and establish cut-off points able to distinguish healthy individuals (control group) from patients with low-grade CIPN G1 (cut-off point 29.5 pg/mL) during treatment monitoring. We also sought to distinguish patients without symptoms of neuropathy (CIPN 0) from those with low-grade CIPN G1 neuropathy (cut-off point 196 pg/mL) and patients without symptoms of neuropathy from those with high-grade CIPN G2 and CIPN G3 neuropathy (cut-off point 218 pg/mL). The areas under the ROC curves (AUCs) obtained in our analysis indicated a high diagnostic sensitivity of NF-L concentration measurements in the assessment of neuropathy among breast cancer patients treated with paclitaxel in NAC therapy. In this way, NF-L concentrations at our cut-off points allowed us to determine the stage of neuropathy with high probability. In addition to breast cancer, the usefulness of NF-L measurements has also been assessed in patients receiving taxanes as part of their chemotherapy to combat malignancies in various locations. In a study of 190 patients with ovarian cancer treated with paclitaxel and carboplatin, serum NF-L levels were observed to significantly increase during chemotherapy. Patients with serum NF-L levels above 150 pg/mL after the first cycle were at risk of developing neuropathy and discontinuing paclitaxel treatment [38].

In another study, serum NF-L levels were measured in 88 patients with non-small cell lung cancer treated with paclitaxel and carboplatin. Following drug administration, NF-L levels increased from baseline 21 days after the first dose of chemotherapy. Similar to our study, NF-L levels were particularly elevated in patients who reported CIPN G2 or CIPN G3 during subsequent treatment cycles. Furthermore, changes in NF-L levels predicted the subsequent development of CIPN G2 and CIPN G3. These results suggest that measuring serum NF-L levels early in paclitaxel and carboplatin therapy may identify patients susceptible to developing higher-grade neuropathy before symptoms appear [14].

It seems that neurofilaments act as specific indicators of axonal damage and may serve as biomarkers for CIPN. Our previous results support the possibility of using NF-Ls in a group of breast cancer patients receiving perioperative chemotherapy. From a clinical perspective, this result provides the opportunity to select patients who should be treated with special attention and implement preventive procedures such as hypothermia during chemotherapy and other methods (acupuncture, exercise, etc.). In some patients, it may also be necessary to switch from paclitaxel to docetaxel or discontinue treatment earlier. NF-Ls could also be used as a biomarker for the implementation of effective pharmacological methods for CIPN treatment.

Our study was based on a unique group of breast cancer patients treated with taxanes in a single-center setting in NAC. However, our study retains some limitations. For example, the number of patient groups was different in each individual study phase (decreasing trend) due to incomplete serum collections during follow-up, especially in the analysis of NF-L levels and the assessment of CIPN severity according to the observations obtained 3–6 months after treatment. The number of patients with CIPN G3 (a clinically relevant group) was also relatively small, due to the severity of symptoms. Therefore, the present study required a combined analysis of patients with CIPN G2 and CIPN G3. There is no other recognized objective parameter available to determine the degree of neurotoxicity. Assessments based on clinical symptoms reported by patients are subjective. Hence, there seems to be an urgent need for further studies to introduce an objective, measurable parameter into clinical practice, depending on the severity of neurotoxicity as a side effect of the treatment.

Limited data from the literature indicate the significant role of NF-L measurement in monitoring neuropathy induced by taxane treatment in patients with breast cancer. Most studies using NF-L concentration measurements focus on neurodegenerative diseases. Previous studies were conducted in small groups of oncological patients. In these studies, the schemes of neurofilament measurements and the designated cut-off points significantly differed. Therefore, there is a need for further studies on NF-L measurements as objective markers of taxane-induced neurotoxicity to develop an optimal scheme for use in oncological practice. In the future, it will also be important to investigate whether NF-L measurements have prognostic significance in predicting the occurrence of peripheral neuropathy among breast cancer patients treated with taxanes in NAC therapy.

We intend to continue the present work in future studies. Firstly, we intend to observe the patients included in this study for a longer period of time. Many publications on CIPN emphasized that among certain groups of patients receiving chemotherapy containing taxanes, CIPN symptoms can appear several weeks or months after the end of chemotherapy [39]. In addition, we are considering further studies on the impacts of neurotoxic chemotherapy regimens among groups of patients with other cancers (gastrointestinal, prostate, and gynecological cancers). Another prospective study will explore the use of CIPN prophylaxis elements (e.g., hypothermia) among a group of patients with breast cancer.

## 5. Conclusions

In this study, we demonstrated the usefulness of NF-L testing as a confirmatory test for early-stage neuropathy, considering that assessments based on clinical symptoms reported by patients are subjective. According to our established cut-off points, serum NF-L levels above 196 pg/mL in breast cancer patients undergoing therapy indicate the presence of low-grade neuropathy, while values above 218 pg/mL may indicate advanced CIPN.

## Figures and Tables

**Table 1:** Clinicopathological characteristics of the patients included in this study.

Parameters	Number of Patients (%)
Age (years) median 50; range 27–77	
Receptor status	
ER+	54 (57)
ER−	40 (43)
PR+	48 (51)
PR−	46 (49)
HER2+	39 (41)
HER2−	55 (59)
Proliferation index (Ki-67)	
Ki-67 ≤ 20	31 (33)
Ki-67 > 20	62 (64)
Ki-67 unknown	1 (3)
Tumor size (T)	
T1	16 (17)
T2	48 (51)
T3	23 (25)
T4	7 (7)
Lymph node status (N)	
N0	47 (50)
N1	35 (37)
N2 + N3	10 + 2 (13)
Distance metastasis status (M)	
M0	94 (100)
Histological grade (G)	
G1	10 (11)
G2	45 (48)
G3	34 (36)
G unknown	5 (5)
Histopathological type	
NOS (not otherwise specified)	83 (88)
ILC (infiltrating lobular carcinoma)	6 (6)
other	5 (6)
Treatment	
4 × ddAC + 12 × PCL	51 (54)
4 × ddAC + 12 × PCL + Carbo + Pembrolizumab	14 (15)
4 × ddAC + 12 × PCL + Carbo	13 (14)
4 × AC + 12 × PCL	7 (8)
4 × AC + 12 × PCL + Carbo	1 (1)
12 × PCL + Carbo + 4 × ddAC + Pembrolizumab	4 (4)
12 × PCL	1 (1)
12 × PCL + Trastuzumab	2 (2)
12 × PCL + Carbo + 4 × ddAC	1 (1)
Biological subtype	
Luminal A	16
Luminal B	12
Luminal B HER2 enriched	27
Non Luminal HER2 positive	1
TNBC/triple negative/	38

**Table 2:** Percentages of patients with CIPN symptoms and median NF-L concentrations, depending on the NAC regimen: PCL vs. PCL plus CARBO.

Chemotherapy Scheme	Medians (Ranges) of NF-Ls (pg/mL) After 3 Cycles of Therapy	Percentage of Patients with CIPN	Medians (Ranges) of NF-Ls (pg/mL) After 6 Cycles of Therapy	Percentage of Patients with CIPN	Medians (Ranges) of NF-Ls (pg/mL) 3–6 Months After the End of Therapy	Percentage of Patients with CIPN	*p*
PCL (+/− AC, trastuzumab)	128 (37.6–568)	35%	268 (14.3–1430)	49%	26.1 (11.4–230)	53%	0.055
PCL+carboplatin (+/− AC, pembrolizumab)	127 (30.9–499)	37%	176 (38.1–881)	58%	22.6 (5.2–85.5)	53%	

**Table 3:** Median NF-L concentrations depending on the severity of CIPN during and after NAC.

Time of Collection of Serum Samples	CIPN Grade							
	G0		G1		G2		G3	
	N	NF-L pg/mL Medians (Ranges)	N	NF-L pg/mL Medians (Ranges)	N	NF-L pg/mL Medians (Ranges)	N	NF-L pg/mL Medians (Ranges)
before starting therapy	87	43.9 (2.8–179)	7	63.1 (36–107)	-	-	-	-
after 3 cycles of therapy	43	131 (30.9–568)	15	99.6 (2.1–268)	7	148 (67.2–475)	1	76 *
after 6 cycles of therapy	29	206 (14.3–567)	15	293 (38.1–1430)	12	246 (43.1–497)	5	662 (367–851)
3–6 months after the end of therapy	19	20.6 (5.2–87.1)	11	35.5 (11.4–230)	6	45.3 (12.7–108)	7	31.4 (17.0–62.8)

* The results of measuring the NF-L concentrations in one patient.

## Data Availability

The data presented in this study are available upon request from the corresponding author due to privacy, legal and ethical reasons.
